# A Spectral-Based Approach for BCG Signal Content Classification †

**DOI:** 10.3390/s21031020

**Published:** 2021-02-02

**Authors:** Mohamed Chiheb Ben Nasr, Sofia Ben Jebara, Samuel Otis, Bessam Abdulrazak, Neila Mezghani

**Affiliations:** 1 Higher School of Communication of Tunis, Research Lab. COSIM, Carthage University, Tunis 2088, Tunisia; mohamedchihab.bennaser@supcom.tn; 2 Laboratoire de Recherche en Imagerie et en Orthopédie, CRCHUM, Montreal, QC H2X 0A9, Canada; samuel.otis.1@ens.etsmtl.ca (S.O.); neila.mezghani@teluq.ca (N.M.); 3 Department of Computer Science, Sherbrooke University, Sherbrooke, QC J1K 2R1, Canada; Bessam.Abdulrazak@usherbrooke.ca; 4 LICEF Institute, TELUQ University, Montreal, QC H2S 3L4, Canada

**Keywords:** Ballistocardiogram signal, connected mattress, spectral features, signal segmentation, human activities classification

## Abstract

This paper has two objectives: the first is to generate two binary flags to indicate useful frames permitting the measurement of cardiac and respiratory rates from Ballistocardiogram (BCG) signals—in fact, human body activities during measurements can disturb the BCG signal content, leading to difficulties in vital sign measurement; the second objective is to achieve refined BCG signal segmentation according to these activities. The proposed framework makes use of two approaches: an unsupervised classification based on the Gaussian Mixture Model (GMM) and a supervised classification based on K-Nearest Neighbors (KNN). Both of these approaches consider two spectral features, namely the Spectral Flatness Measure (SFM) and Spectral Centroid (SC), determined during the feature extraction step. Unsupervised classification is used to explore the content of the BCG signals, justifying the existence of different classes and permitting the definition of useful hyper-parameters for effective segmentation. In contrast, the considered supervised classification approach aims to determine if the BCG signal content allows the measurement of the heart rate (HR) and the respiratory rate (RR) or not. Furthermore, two levels of supervised classification are used to classify human-body activities into many realistic classes from the BCG signal (e.g., coughing, holding breath, air expiration, movement, et al.). The first one considers frame-by-frame classification, while the second one, aiming to boost the segmentation performance, transforms the frame-by-frame SFM and SC features into temporal series which track the temporal variation of the measures of the BCG signal. The proposed approach constitutes a novelty in this field and represents a powerful method to segment BCG signals according to human body activities, resulting in an accuracy of 94.6%.

## 1. Introduction

Amidst the expansion in the field of the Internet of Medical Things (IoMT), a significant shift in the paradigm of tele-healthcare has been recorded. In particular, the monitoring of vital signs is gaining attention and has established itself as no longer a commodity but a necessity in this field. Four principal vital signs serve to establish an early warning score, namely the body temperature, blood pressure, heart rate (RR) and respiratory rate (RR). This score is the gold standard when it comes to quantifying the degree of illness of patients [1,2].

The Ballistocardiogram signal (BCG) is a noninvasive, unobstructive measure of the ballistic force generated by the circulation of the blood in the body. Several sensors have been designed to extract the BCG signal using for instance cameras [3], accelerometers [4], weight scales [5,6], ear wearables [7] and piezoelectric sensors [8]. Recently, an interesting microbend Fiber Optic Sensor (FOS) has been placed under mattresses; this noninvasive and unobstructive method is boosting the use of the BCG in telemedicine [9] since it provides more flexibility and eliminates the inconveniences present in invasive or minimally invasive methods [10].

Several works have studied the extraction of vital signs from BCG signals. For instance, in [11], the authors developed different methods for the extraction of the RR such as the Harmonic and Noise (HNM) model and Wavelet Transform (WT), yielding promising results. In [12], the authors proposed a machine learning-based approach for the estimation of the beat-to-beat HR from a highly volatile BCG signal. Features describing the BCG waveform were extracted, and a modified *k*-means algorithm was used as an inference tool. Other works, such as in [13], implemented a template matching approach to detect the J-peaks of the BCG waveform. In [14], two different methods were implemented for the detection of the HR, namely the Maximum Overlap Discrete Wavelet Transform (MODWT) and Complete Ensemble Empirical Mode Decomposition with Adaptive Noise (CEEMDMAN) methods.

However, the previous works considered signal segments with preset properties and characteristics that describe perfect conditions (a subject laying on the mattress and breathing normally) and are thus distant from the real-life challenges when real data are processed. Indeed, these challenges are present due to the manifestations of spontaneous or illness-related activities of the subject during the recording of the signal. Examples of these instances include movement, coughing, holding breath, etc.

The main focus of this paper is to extend the systems developed in previous works to measure HR and RR by including a preprocessing stage with a set of classification and segmentation tools to decide which portions of the BCG signal, HR and RR are possible to measure. The full approach also allows the precise determination of the start and the endpoints of different kinds of human-body activities that can be extracted from the BCG signal.

**Figure 1:**
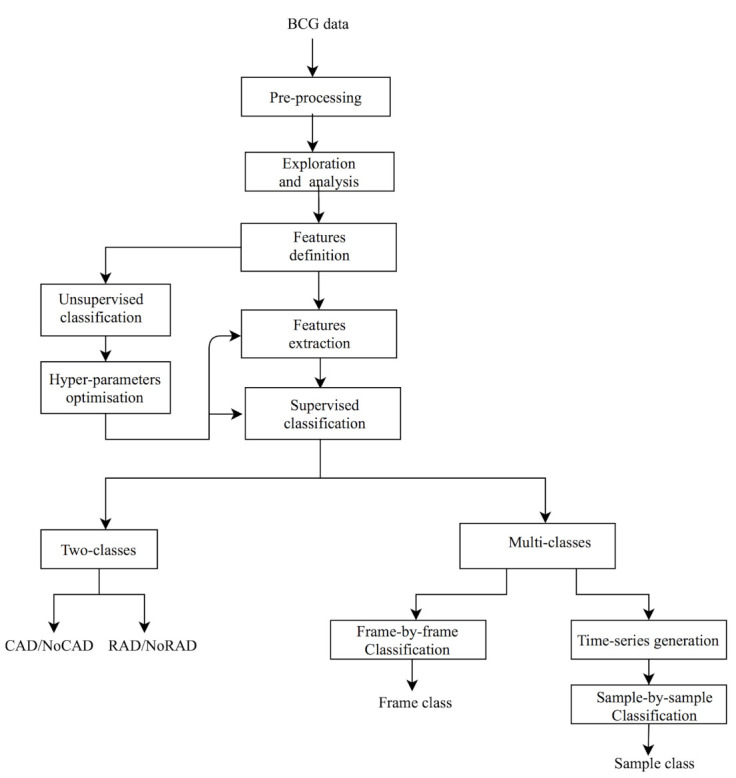
Flowchart of adopted methodology. BCG: Ballistocardiogram. CAD: cardiac activity detection; RAD: respiratory activity detection.

We adopted a progressive approach, the flowchart of which is presented in Figure 1. The first step, after the BCG signal pre-processing, of content-based BCG signal exploration and understanding includes the temporal and spectral analysis of the BCG signal during different human body activities (for example, resting, coughing and movement). The analysis permits us to consider the use of two spectral features, namely the Spectral Flatness Measure (SFM) and Spectral Centroid (SC). These are defined according to a frame-by-frame analysis, which means that the BCG signal can be decomposed into pre-defined time duration slices. After features behavior analysis, a clustering task, which is the core of many segmentation methods, is carried out in order to determine the usefulness of selected features for classifying the BCG signal into many classes. The number of classes and the hyper-parameters related to the frame size are optimized thanks to the Gaussian Mixture Model (GMM). Next, in order to establish the link between the number of classes and the nature of human body activity, a supervised classification is proposed. Two types are defined: the first one is a two-class approach that aims to classify a selected frame into one permitting the measurement of HR (binary flag cardiac activity detection (CAD)/NoCAD as an output) and another one permitting the measurement of RR (respiratory activity detection (RAD)/NoRAD). We recall that this kind of binary output is useful to drive HR and RR detectors. In fact, during coughing, there is no need to try to measure the RR, for example, since information on respiratory activity is absent and not relevant. The second supervised classification is a refined one since it gives information about the type of human body activity (resting, coughing, expiration, movement, etc.). We deal with coarse and fine approaches. The coarse approach makes use of features calculated for each frame while the fine approach considers a sample-by-sample classification. To this end, frame-by-frame features are converted to time-series thanks to a novel idea presented in this paper. The segmentation consists of identifying the bounding of each human body activity.

## 2. Material and Methods

### 2.1. Data Collection

#### 2.1.1. Acquisition Process

The project was carried out at the Centre Hospitalier de l’Université de Montréal (CHUM) in Montréal, Canada. The protocol was accepted by the institutional ethics committees of the Ecole des Technologies Supérieures (ETS), the TELUQ university, the University of Sherbrooke and CHUM.

The study was conducted on healthy subjects, comprising three males and three females, aged between 20 and 35 years, in good health and without known cardio-respiratory disorders. Once the subjects agreed to participate in the study, information concerning their demographic and anthropomorphic details (age, weight, height, etc. ) were collected.

**Figure 2:**
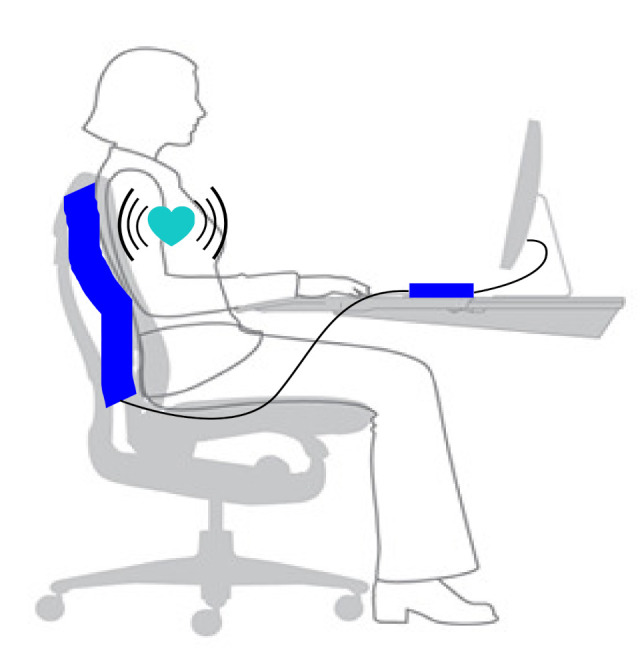
Illustration of the experimental protocol activities [16].

The system used for collecting data included a small FOS mattress and a module to gather optical data from the mattress. This particular process and more details about the feasibility, conception and performance of the FOS micro-bend sensors are available in the literature (for example, [11] or [15]). The FOS mattress was fixed on the back of a regular office chair, as shown in Figure 2. The system used for collecting data included a small FOS mattress and a module to gather optical data. The raw data were sampled at 50 Hz by the module. During the whole experiment, the subject wore a Hexoskin and was asked to sit still on the chair for a duration of 5 min. During this time, a Raspberry Pi 3B recorded the data streamed on the serial port of the FOS’s module.

#### 2.1.2. Experimental Protocol

To simulate common human body activities, subjects were asked to obey a certain experimental protocol composed of the following steps: (1) normal breathing for 30 s, (2) coughing three times, (3) normal breathing for 300 s, (4) holding breath for 30 s, (5) expiration, (6) normal breathing for 60 s, (7) coughing 10 times with a between-cough interval of 5 s, (8) normal breathing for 30 s, (9) coughing 10 times with a between-cough interval of 2 s, (10) normal breathing for 120 s, (11) standing up, (12) sitting down, (13) steps 10 and 11 repeated 4 times and (14) standing up.

Thus, aside from remaining in a still position, other human body activities that commonly occur and can alter vital sign measurements from BCG signals were introduced. We explored (i) coughing as a frequent activity executed by the human body, which induces a certain internal disturbance in the respiratory and cardiac rhythms; (ii) holding one’s breath as another activity that renders respiration activity impossible to consider while cardiac activity is possible to extract; and (iii) standing up and sitting down as an example of movement types.

**Figure 3:**
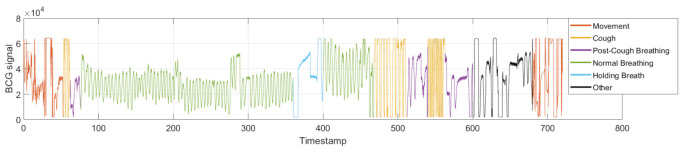
Illustration of the BCG signal during the experimental protocol activities. Each color corresponds to a human body activity.

Figure 3 illustrates the BCG signal during the experimental protocol activities. This is discussed in detail later.

The ground truth—i.e., the human body activities—was created manually by experts who adjusted the frontiers so that a precise ground truth was created. This label information was used later in frames and then in spectral features.

### 2.2. Ballistocardiogram Signal Description

#### 2.2.1. Cardiac Information

**Figure 4:**
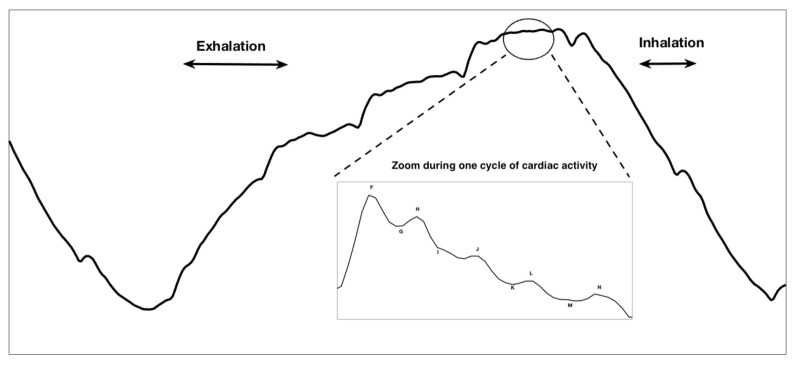
Theoretical respiratory and cardiac activities present in the BCG waveform.

The BCG signal is a measure of the acceleration of blood through the veins. In broader terms, it is a measure of the ballistic force generated by the heart. For a person placed on a bed with minimal friction and minimal movement artifacts, one cycle of ideal cardiac activity appearing in BCG signal is illustrated in the enlarged part of Figure 4. This contains eight different waves annotated as *F*, *G*, *H*, *I*, *J*, *K*, *L* and *M*. They are divided into three groups: pre-ejection (F,G,H), ejection (I,J,K) and diastolic (L,M,N). The *F* wave (rarely seen in the BCG representation) is closely related to pre-systolic events of the heart. The *G* wave corresponds to a trough preceding the systolic waves. The *H* wave is the first one in the systolic cycle; it corresponds to the maximum peak recorded (which is synchronous to the isovolumetric contraction) [17]. *I*, *J* and *K* waves are the most recognized waves in the BCG, occurring during the systole. The *I* wave simulates the acceleration of blood in the ascending aorta and pulmonary arteries; the *J* wave is the main positive wave occurring in the systole, simulating the acceleration of the blood when going through the second part of the aorta; and the *K* wave occurs before the end of the systole. *L* and *M* are the diastolic waves that represent headwards deflections following the *K* wave [18,19].

#### 2.2.2. Respiratory Information

**Figure 5:**
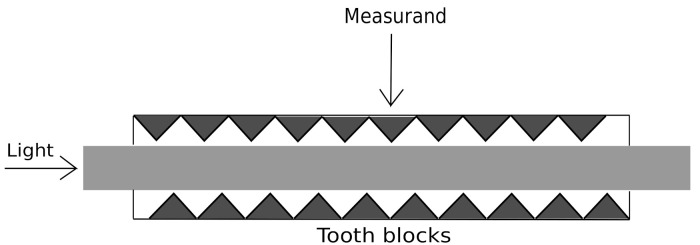
Microbend FOS principle. The light passing through the microbend FOS is modulated by the deformations in the optical fiber due to the displacement of the micro-benders [23].

It is worth mentioning that the described waveform depends greatly on the used acquisition system. In fact, multiple sensors using different electrical, optical or mechanical devices are currently available [20]. For example, optical fiber-based systems extract the aforementioned waveform, but it is believed that the resulting signal presents a respiratory component which corresponds to the movement of the thoracic cage of the patient [11,21]. In fact, the BCG signal studied in this work was acquired using a microbend FOS which provides a new way of acquiring the mechanical activity of the human body. More precisely, we used a Juvo’s skin non-contact feature, benefitting from the patented invention of fibre-optic sleep and vital signs sensors [21,22]. The BCG signal formation was achieved through the intensity attenuation of the light passing through an optic fiber in response to a mechanical stimulus on the fiber, as shown in Figure 5.

The FOS sensor, typically placed at the level of the thoracic cage, captures the longitudinal BCG as well as the inspiration and expiration of the body [23]. In fact, during the inspiration phase, the inhalation of air by the patient causes the appearance of a downward trend in the BCG signal, which corresponds to the increase of the force executed on the sensor placed behind the thoracic cage of the body. During the expiration, the exhalation of the air in the body of the patient causes a decrease in the force on the sensor, leading to an upward trend. The coupling of these upward and downward trends constitutes the general respiration waveform of the BCG [21,24]. An example of the respiratory cycle present in the BCG signal acquired with the FOS system is illustrated in the upper part of Figure 4.

### 2.3. Spectral Features for the Identification of Various Activities

#### 2.3.1. Spectral Features Motivation

The cardiac and respiratory activities were repeated periodically. The term x(t) denotes any one of the periodic signals generated in the BCG signal to describe the cardiac or the respiratory activity; according to its periodicity property, its Fourier transform is a sum of Dirac pulses equally spaced in the frequency axis at frequencies that are multiples of $f_{0}=\frac{1}{T_{0}}$:

(1) $$X\left(f\right)=\sum_{k\in Z}X_{k}\delta \left(f-kf_{0}\right),$$

where $X_{k}$ is the $k^{th}$ Fourier transform coefficient, calculated during one period according to the following formula:

(2) $$X_{k}=\frac{1}{T_{0}}\int_{\frac{-T_{0}}{2}}^{\frac{T_{0}}{2}}x\left(t\right)e^{-j2\pi \frac{k}{T_{0}}t}dt.$$

Thus, a periodic signal can be represented as a sum of sine waves, and thus the Fourier transformation of this particular signal is spiky.

We used the two features of the Spectral Flatness Measure (SFM) and Spectral Centroid (SC). They are computed as follows. First of all, the discrete-time BCG signal, denoted as x(n), is decomposed into frames of short duration. These frames should be long enough to carry information about the activity but not overly long to avoid an overlap of two or more different activities. In the frequency domain, the short-term Fourier transform is calculated and its amplitude is extracted. Its module is denoted as |X(m,k)|, where *m* is the frame index and *k* is the discrete frequency.

#### 2.3.2. Spectral Flatness Measure (SFM)

This is also known as Wiener entropy [25]. It is a signal processing measure used to describe the flatness of the spectrum of a certain signal, defined as the ratio of the geometric mean and arithmetic mean of the spectrum:

(3) $$SFM\left(m\right)=\frac{\sqrt[{N}]{(}\prod_{k=1}^{N}\left|X\left(m,k\right)|\right)}{\frac{\sum_{k=1}^{N}\left|X\left(m,k\right)\right|}{N}},$$

where *m* is the frame number, *k* is the frequency bin index and *N* is the number of frequency bins.

If the spectrum of the frame is flat, SFM(m) will be close to 1. In the particular case of white noise, the spectrum is constant, and thus the SFM(m) value is equal to one. The SFM is mostly used in audio-related fields, for instance. For example, we relate the detection of voiced and unvoiced speech frames [25], the analysis and recognition of whispered speech [26] and emotional speech synthesis and transformation [27]. However, few works have implemented such features in the framework of vital signals, which makes the subject of this work a novelty. In fact, we believe that the pertinence of this feature is supported by the intuition of their physical significance.

#### 2.3.3. Spectral Centroid (SC)

The Spectral Centroid indicates the location of the center of mass of the spectrum. It is defined as follows:

(4) $$SC\left(m\right)=\frac{\sum_{k=1}^{N}\left|X\left(m,k\right)\right|.f\left(k\right)}{\sum_{k=1}^{N}\left||X\left(m,k\right)\right|},$$

where f(k) is the frequency in Hertz related to the frequency bin *k*. Once again, because the Spectral Centroid is a good predictor of the “brightness” of a sound, it has been widely used in digital audio and music processing, such as musical genre classification [28]. To the best of our knowledge, its use for a BCG signal is also a novelty.

### 2.4. Unsupervised Classification for the Exploration of the Number of Activity Classes

We conducted an exploratory data analysis in order to determine the different classes of activities in the signal. First, a general description of the available features and their modeling process in an unsupervised manner was made. No prior knowledge about the available BCG signal was taken into consideration. This justified the existence of different classes and permitted us to define the hyper-parameters that were useful for the effective classification.

#### 2.4.1. Features Histograms and GMM Modeling

The Gaussian Mixture Model (GMM) is used for unsupervised learning algorithms to model clusters of points. Each cluster is assigned a Gaussian function where the mean represents the center of the cluster and the variance describes the spread within it. We denote $F\left(m\right)={\left[SFM\left(m\right),SC\left(m\right)\right]}^{T}$ as the two-component feature vector of the frame *m*. The distribution can be modeled using the GMM as follows:

(5) $$P\left(F|\mu ,\Sigma \right)=\sum_{k=1}^{K}\frac{\pi_{k}}{2\pi |\Sigma_{k}{|}^{1/2}}exp\left(-\frac{1}{2}{\left(F-\mu_{k}\right)}^{T}\Sigma^{-1}{\left(F-\mu_{k}\right)}^{T}\right),$$

where *K* is the number of clusters (number of Gaussian components), $\pi_{k}$ is the weight of the $k^{th}$ Gaussian (where the sum of all of them is equal to one), $\mu_{k}$ is the mean, $\Sigma_{k}$ is the variance and $|\Sigma_{k}|$ is its determinant.

Classically, and regardless of the classification problem, many criteria are defined to formally determine the number of Gaussian components; for example, the Akaike Information Criterion (AIC) [29] and Bayesian Information Criterion (BIC) [30]. They are defined as follows:

(6) AIC(K)=2K−2log(ML),

where ML is the maximum likelihood of the model.

The Bayesian Information Criterion introduces a penalty term,

(7) BIC(K)=Mlog(ML)−2log(ML),

where *M* is the number of training examples. Similarly to AIC, low values of BIC indicate a good fit of the model.

#### 2.4.2. Frame Size Optimisation

GMM modeling requires the optimization of the frame size. The used criterion is the silhouette, which provides information about the goodness of fit of a certain model [31]. It takes into consideration the between-cluster distance as well as the within-cluster distances, which makes it particularly interesting for an unsupervised learning context. The expression of the silhouette s(i) is the following:

(8) $$s\left(i\right)=\left\{\begin{array}{c} \frac{b(i)-a(i)}{max(b(i),a(i))}\;if\;card\left(C_{i}\right)>1\\ 0\;\;if\;card(C_{i})=1, \end{array}\right.$$

where a(i) is the within-class distance, b(i) is the between-class distance, $C_{i}$ is the $i^{th}$ cluster and card(.) is the cardinal operator. a(i) and b(i) are computed as follows:

(9) $$a\left(i\right)=\frac{1}{card(C_{i})-1}\sum_{j\in C_{i},i\neq j}d\left(i,j\right),$$

and

(10) $$b\left(i\right)=\min_{k\neq i}\frac{1}{card(C_{k})}\sum_{j\in C_{k}}d\left(i,j\right),$$

where d(i,j) is the distance between the points *i* and *j*.

After calculating s(i) for each frame under different frame sizes, the mean value is determined for each frame size.

### 2.5. Supervised Binary Classification for Vital Signs Detection

Supervised classification was performed using the K-Nearest Neighbors (KNN) algorithm thanks to its simplicity and as we knew that other classification techniques have been tested and have shown poorer or similar performances. The KNN algorithm is a non-parametric method that is used for classification. The variant adopted was Fine KNN, which is the finest variation of KNN since it labels the new input with the same label as its nearest neighbor. The BCG signal frames were classified based on their pertinence to detect the heart rate and the respiratory rate. In this regard, all frames were labeled into one of the following activities: cardiac activity detection (CAD) or no cardiac activity detection (NoCAD) and respiratory activity detection RAD or no respiratory activity detection (NoRAD).

### 2.6. Multi-Class Classification and Segmentation

The BCG signal frames were then classified according to human body activities. Seven classes were used: normal activity in still position, coughing, post-coughing, holding breath, expiration, movement and others. Two different classification approaches were adopted. First, a classification was made for each frame, which is called frame-by-frame classification. The second approach was used for each sample and is called sample-by-sample classification.

Enframing is a classical method in signal processing, and it refers to splitting the BCG signal into temporal segments. For each frame, features are extracted, and decision-making about classification is related to the whole segment.

**Figure 6:**
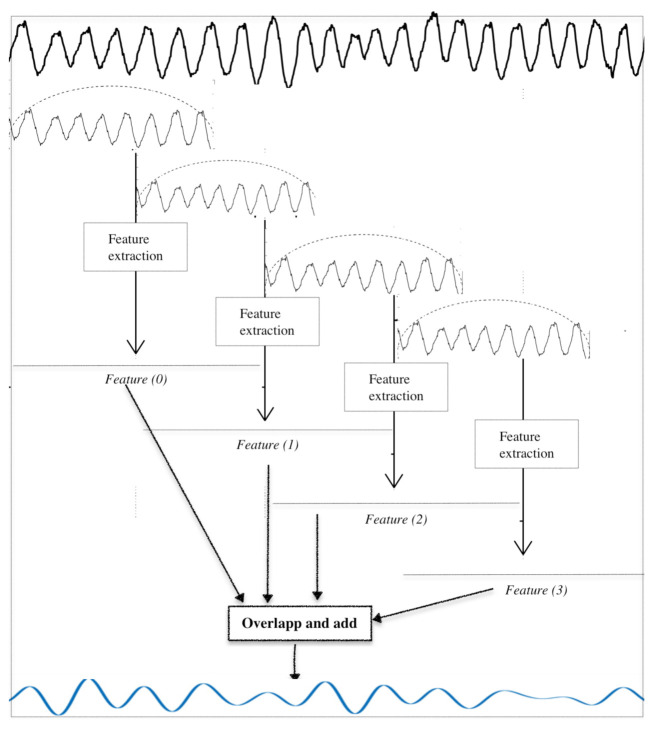
Steps of time-series generation.

The approach used for sample-by-sample classification was as follows. Instead of using raw values of SFM and SC for each frame and risking the resulting limitations (coarse decision for long-duration frame, boundary epochs, etc.), we created time-series out of the SFM and SC values computed for each frame. The adopted approach is described below; it is also illustrated in Figure 6.

-  First, the original BCG signal was decomposed into frames of length 1024 with an overlap of 960 samples (an increase of 64 from frame to frame). The Hamming window was used for this. An example of the original BCG signal, the moving Hamming window and the generated frames are illustrated in the top part of Figure 6.
-  The features, namely SFM(m) and SC(m), were extracted for each frame.
-  Each scalar feature SFM(m) (resp. SC(m)) was used to construct a “sub-time-series”. This latter was a constant vector, whose value was equal to SFM(m) (resp. SC(m)) and whose length was equal to the frame size. The constant sub-series are shown in Figure 6 below those of previous steps (horizontal lines labelled Features(m)).
-  The whole set of sub-series was put together at the input of an “overlap and add module”. This step was equivalent to the inverse of windowing and frames decomposition; the aim of this process was to construct time-series from features that represented the spectral content of the BCG signal. Their length was equal to that of BCG signal, and they are denoted as $sfm_{signal}$ and $sc_{signal}$, respectively, for the Spectral Flatness Measure and Spectral Centroid.
-  The classification was then conducted on each sample of the new time-series. This process is called sample-by-sample-classification.

### 2.7. Evaluation Metrics

During this study, some common classification criteria were used. Due to a lack of space, only some of them are given here. In case of binary classification, we mainly retain the True Positive Rate (TPR) and the Positive Predicted Value (PPV). The TPR, called also sensitivity or recall, measures the proportion of detected positives from the actual positive (true positives and false negatives) while the PPV measures the proportion of detected positives from the real positive.

In the binary classification, the provided confusion matrix contains the TPR and the PPV values on the diagonal, while the complementary metrics define the False Discovery Rates (FDR) and the False Omission Rates (FOR). The generalized formulas of the evaluation metrics are included below:

(11) $$TPR=\frac{true\;positives}{true\;positives+false\;negatives}.$$

(12) $$PPV=\frac{true\;positives}{true\;positives+false\;positives}.$$

In case of multiple classes (more than two), the confusion matrix in terms of TPR and PPV is used. Each term of the matrix is written as follows:

(13) $$Conf_{TPR}\left(i,j\right)=\frac{M_{ij}}{\sum_{j=1}^{L}M_{ij}},$$

where $M_{ij}$ is the number of predictions of class *i* that belong to class *j*, *L* is the number of classes. The PPV confusion matrix is given by

(14) $$Conf_{PPV}\left(i,j\right)=\frac{M_{ij}}{\sum_{i=1}^{N}M_{ij}}.$$

## 3. Results and Discussion

### 3.1. Illustration of Temporal and Spectral Properties

#### 3.1.1. Overall Temporal Evolution

Figure 3 illustrates an example of the BCG signal acquired when following the experimental protocol. The different human body activities are plotted in different colors. The objective of this is to highlight the differences in the BCG signal according to the activity; for example, during normal respiration, the signal periodicity is observable, and so it is thought that we can easily measure the cardiac and respiratory vital signs using classic approaches. During the coughing exercise, it was difficult to detect the periodicity that originated from the respiration rhythm and the cardiac rhythm. In fact, the recording of the BCG shows that the signals attained the maximum value allowed by the acquisition equipment. This can also be noted during the movement phase. The peaks displayed in these different activities were not a result of the physiological activity of the body but of the physical activity. Moreover, in addition to the classes defined in the experimental protocol, a post-cough class has been added. Indeed, careful observation of this step showed particular variations in the BCG signal which did not appear with the other classes. Finally, another class called “others” includes other behaviors not foreseen in the experience.

To conclude, this particular representation, based on manual segmentation, highlights the importance of separately detecting the different activities in the BCG signal. The temporal and spectral contents of the BCG signal during some kinds of activities are detailed below.

#### 3.1.2. Illustration during Quiet Activities

**Figure 7:**
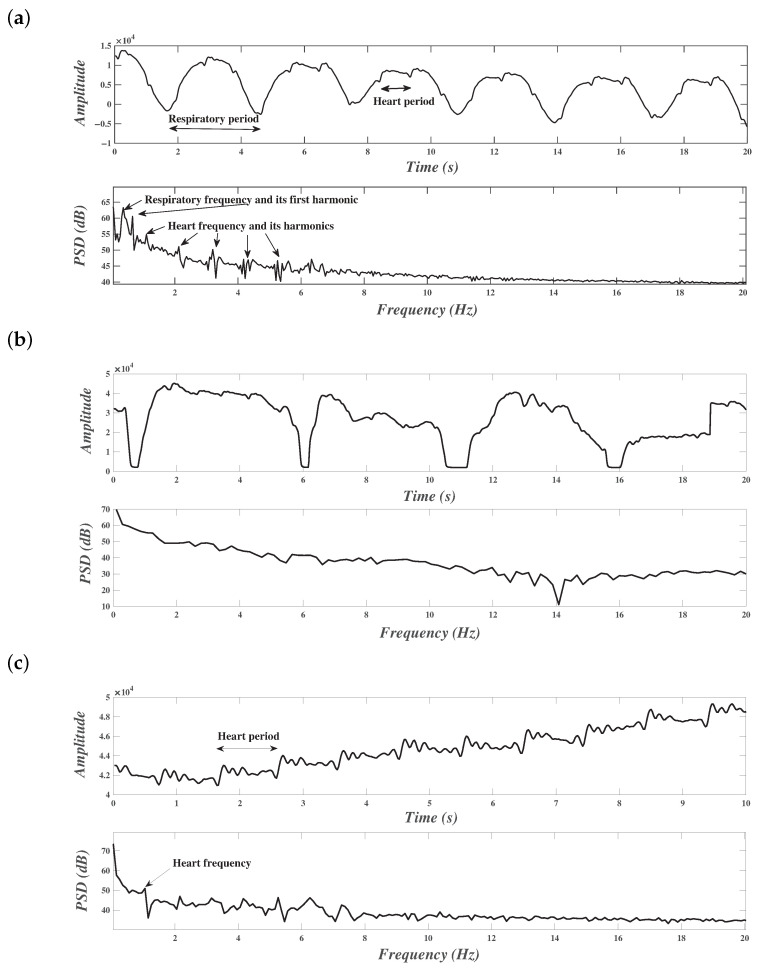
Illustration of temporal evolution and frequency representation of BCG signals. (**a**) normal respiration in still position, (**b**) during coughing activity, (**c**) while holding breath.

The resulting BCG signal acquired using a microbend FOS during normal activity in still position contained the cardiac waveform enriched with the respiration waveform. The top part of Figure 7a illustrates the temporal evolution of a BCG signal during this kind of activity. One can notice the periodicity of cardiac activity as well as that of respiratory activity. The respiratory periodicity is related to the large period appearing in the BCG signal. The cardiac periodicity is less than the respiration activity. In the frequency domain (bottom part of Figure 7a), one can see the presence of different spikes; they correspond to the respiratory frequency and its harmonics and to the cardiac frequency and its harmonics. In fact, the Fourier transform of a periodic signal is a sum of Dirac pulses equally spaced in the frequency axis at frequencies multiple of the fundamental frequency (see for example [32]). The respiratory frequency appears at the first spike; its first harmonic is the second one rising in the spectrum. However, higher-order harmonics do not appear because they are not relevant. On the other hand, the cardiac harmonics are relevant, and many of them rise significantly in the spectrum.

It is important to note that the values of cardiac and respiratory frequencies are validated by the available ground truth. In fact, in this study, we used the clinically validated Hexoskin intelligent textile. This was embedded with sensors giving accurate measurements [33,34]. It is also worth mentioning that more sophisticated methods can be used to extract these vital signs, such as the Wavelets Transform [35], discrete Fourier transformations [36], empirical mode decomposition [37] and many others (for example, [11]). These methods are, however, beyond the scope of this work.

#### 3.1.3. Illustration during Special Activities

The former illustration describes a perfect environment as the subject remained in a still position. This situation is not always present because of the subject’s movement; thus, it is important to study the BCG signal behavior during various human body activities to determine if the BCG signal continues to manifest cardiac and respiratory vital signs.

The next subject activity studied was coughing. During this test, the subject was asked to cough while the BCG signal was acquired. This experience was studied as we believed that coughing activities reduced the reliability of extracting both vital signs. Furthermore, it was enlightening to capture the coughing frequency to establish information about the medical state of the patient. This application is promising especially in the context of SARS-CoV-2, where the coughing frequency can be an indicator of infectious disease [38].

Figure 7b represents the temporal evolution of the BCG signal (on the top) as well as the frequency representation (on the bottom) during four cough cycles. One can notice the existence of valleys with constant values due to the explosive part of the cough, which led to saturation in the lowest values. These valleys alternated with shapes that can be found in the BCG signal of normal activity. However, the periodicity induced by the respiratory activity was absent. On the other hand, a form of periodicity due to cardiac activity may exist, but it was challenging to accurately extract the corresponding waveform. From frequency representation, the spikes due to normal respiration disappeared. This fact calls into question the possibility of extracting vital signals during coughing.

Another activity performed by the subject was holding their breath. The subject was asked to hold their breath for a fixed amount of time. It was believed that the act of holding breath would nullify the effect of inspiration and expiration, and thus the effect of the respiratory activity. A temporal sequence and its frequency representation are displayed in Figure 7c. The signal portion contains only the cardiac information which allows the measurement of the heart rate.

According to these described situations, and to others that will be described later, it was established that measuring the heart and respiratory signs from BCG signals at all times is challenging and not possible in certain cases. It is thus crucial to carry a study that identifies relevant kinds of body activities in order to determine the possibility of vital signs measurement.

### 3.2. Illustration of Spectral Features

To illustrate, in a preliminary stage, the effectiveness of SFM and SC to discriminate between different kinds of activities that appear in the BCG signal, the following steps were followed. The signal was decomposed into frames of 20 s duration. This choice was justified as follows: the respiratory cycle is longer than the cardiac cycle, and techniques to measure the longest rate—i.e., the respiratory rate—require the existence of at least three respiratory cycles in the frame. Knowing that the lowest respiratory rate (highest period) is 10 respiration cycles per minute, three cycles were obtained during $3\frac{60}{10}=18\;s$. We chose a duration of 20 s, giving 1000 samples for a sampling frequency of 50Hz. It is important to note that this duration seemed to be long enough that boundary epochs (i.e., the time intervals for a transition between one body activity and another occurring) are handled. Unfortunately, these epochs are considered in the spectral features for frame-by-frame analysis, but thanks to the sample-by-sample analysis carried at the end of this study, this limitation was alleviated.

After framing, a Fast Fourier Transform FFT with a length of 1024 (the nearest power of two to 1000 samples) was applied and its module was extracted. Next, SFM and SC features were calculated according to Equations (3) and (4).

#### 3.2.1. Scatter Plot

**Figure 8:**
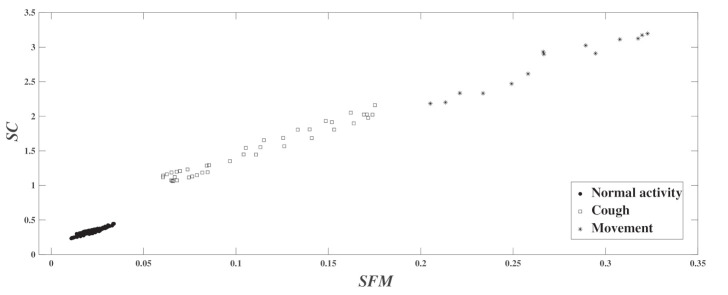
Distribution of the Spectral Flatness Measure (SFM) and Spectral Centroid (SC) values in the dataset.

Figure 8 represents a scatter of these features for the still position, during coughing and during movement. One can notice distinct regions, confirming the ability of these features to discriminate between various activities. Each activity was described by a cluster of points. Moreover, the cluster of the (SFM, SC) pair showed lower values for quiet activity when compared to coughing. Moreover, motion seemed to increase the feature range values. In fact, Figure 8 illustrates the existence of certain decision boundaries to separate between pertinent frames and noisy frames (normal vs coughing and movement) but also a decision boundary to separate between the three classes since movement activities seemed to have a wider range of SFM values.

#### 3.2.2. Features histograms and GMM modeling

**Figure 9:**
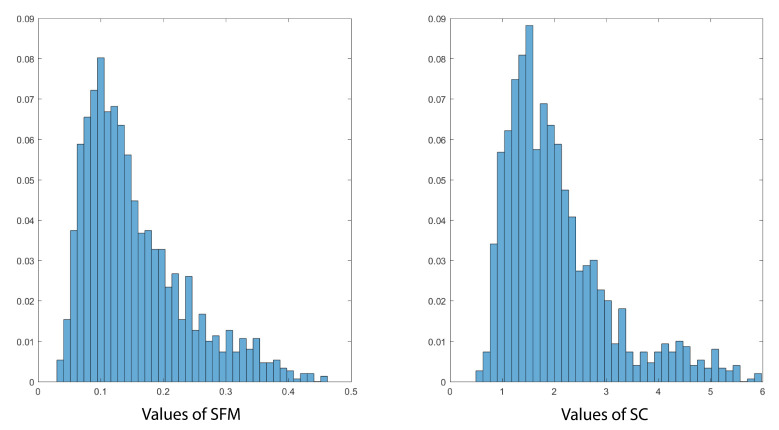
Histograms of SFM and SC values in the dataset.

Figure 9 shows the histograms of the selected features, namely SFM and SC. A total number of 1500 frames with a length of 1024 were obtained from the dataset. From Figure 9, one can notice the existence of two regions: the first one has low values of SFM and SC and the second one has greater values. The previously established remarks support the hypothesis that the first region corresponds to frames representing quiet physical activity. In these frames, cardiac and respiratory activities are relevant. In opposite, the second region, the higher values of SFM and SC correspond to human activity (movement) or physiological activities (coughing, holding breath, expiration, etc.).

#### 3.2.3. Hyper-Parameter Optimization in Unsupervised Classification

**Figure 10:**
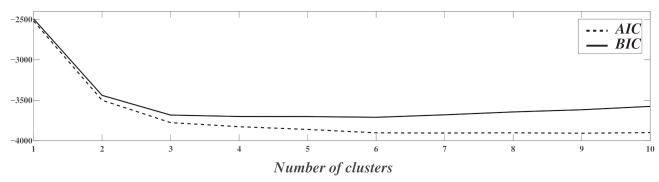
Representation of the Akaike Information Criterion (AIC) and Bayes Information Criterion (BIC) as a function of the number of Gaussian components.

Figure 10 shows the evolution of AIC and BIC as a function of the number of components in the GMM. This number infers the sub-optimal number of classes that can be adopted in the presented BCG signal. The most significant slope (in magnitude) matches the two classes benchmark. This supports the adoption of two classes if the two different classes should be interpreted as those of still activity and moving activity. If more refined classes are needed, Figure 10 shows that the optimal number of classes is around six or seven, as this corresponds to the minimum of the AIC and the BIC representations. This corresponds to the number of activities done by the subject and thus confirms the isolation of the post-cough activity from normal respiration, which corresponds with the theory of multi-labeling [39].

#### 3.2.4. Frame Size Optimisation

**Figure 11:**
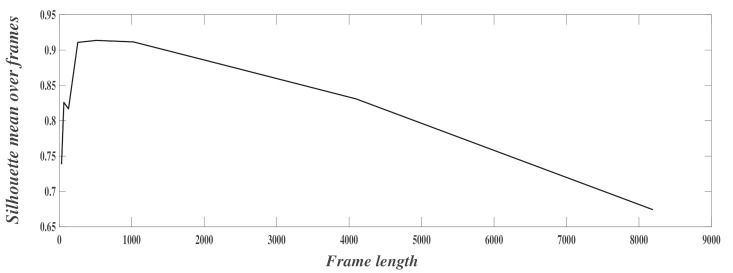
Variation of the mean of silhouette over the frames length.

In the previous experiments, the size of the frame was chosen equal to 1024 samples (corresponding to 20.48 s). This was chosen as an example to describe the determination of the number of classes. The next experiment aimed to optimize the frame size. Figure 11 shows the evolution of the mean value of s(i) versus the frame length. For high frame lengths, the model performs badly since frames can represent different states (different human body activities). The same was observed for very low frame lengths since short frames do not contain enough representative information to characterize the activities. The best trade-off was obtained for frame lengths of 512 or 1024. Note that the tested values are a power of two since the Fast Fourier Transform is applied on frames to compute the features.

### 3.3. Binary Supervised Classification Results

The first positive class, including quiet activity, post-coughing and holding breath, refers to the possibility of extracting the heart rate from the corresponding frames. The negative class includes movement and coughing. The classification results (in percentage) are presented in Table 1. The obtained results show the very good detection of the CAD class. However, a mis-classification of 33% for NoCAD is observed. This means that roughly a third of NoCAD is classified as relevant to measuring the heart rate. This result supports the idea that cardiac vitals can be extracted from coughing activities, which also explains the results obtained in Figure 8 where the values of SFM and SC occupy different regions for coughing and movement activities. It also indicates the presence of a cardiac component in the coughing activity compared to movement.

Comparing the TPR and PPV values in the confusion matrix, one can observe that the values representing the refinement of the detection of the model show good results for the negative class (85%); this confirms the previous hypothesis stating that certain activities classified as noisy represent cardiac features.

Next, dealing with binary classification based on respiratory rate detection, the first positive class including quiet activity refers to the possibility of extracting the respiratory rate from the corresponding frames. The negative class includes movement, holding breath and coughing. The classification results (in percentages) are presented in Table 2.

Similarly to the case of cardiac classification, good classification results were obtained in terms of the detection of the pertinent frames. However, a mis-classification of 28% in the class of NoRAD was recorded. This suggests the possibility of the extraction of the RR from the activities labeled as NoRAD. A possible explanation of this phenomenon is the possibility of the detection of the RR from other activities since this latter may contain the pertinent I,J,K sub-wave of BCG cycle (illustrated previously in Figure 4). In fact, the process of detecting the RR from the cardiac waveform is well established in the literature with the ECG signal. This latter contains the QRS complex commonly used to measure heart rate [40]. The obtained results support the possibility of the existence of similar extractions in the I,J,K complex.

### 3.4. Supervised Muti-Classes Results

The multi-class supervised classification corresponded to the seven classes describing major human body activities. They were as follows: normal activity in still position, coughing, post-coughing, holding breath, expiration, movement and others. The results in terms of TPR and PPV are represented in Table 3 and Table 4 for the case of frame-by-frame classification. The obtained classification accuracy was 75.5%.

The results described in the TPR and PPV confusion matrices show mis-classifications in the class of coughing in terms of the TPR. This mis-classification means that the deployed model classifies all the coughing activities correctly but struggles to detect this class. This is shown by the 100% PPV result compared to only 38% for TPR. This particular interpretation inspired the use of $sfm_{signal}$ and $sc_{signal}$ since the process of creating such signals places more emphasis on the temporal evolution of the features and thus serves to detect the coughing class. Mis-classifications between the classes of holding breath and normal respiration present in the PPV matrix are intuitive since both classes present periodicity and represent one of the downsides of the adopted features. Mis-classifications in the class of expiration are mostly due to the availability of samples. We believe that the expiration class is underrepresented in this work, and more sophisticated methods for unbalanced classification such as one class classifiers [41] or model weights [42] can be adopted. However, this is beyond the scope of this research.

In this part, the sample-by-sample classification results are shown. Table 5 and Table 6 show the classification results of the adopted model. First, we can see an improvement in the detection of the coughing activity, as we achieved a TPR of 92% while utilizing the previously described signals. This supports the utility of generating $sfm_{signal}$ and $sc_{signal}$. The mis-classification between the holding breath and normal respiration is still present, and this supports the previously established explanation and the lack of the adopted features in terms of distinguishing between these two classes. The overall classification accuracy using the sample-by-sample approach is 94.6%, which represents a huge improvement compared to the previously recorded 75.5%.

## 4. Conclusions

In this work, we proposed an approach to generate two binary flags indicating the useful frames permitting the measurement of cardiac and respiratory rates from a BCG signal. We have also presented a refined BCG signal segmentation technique according to the phenomena that occur in the process of recording the BCG signal. These described occurrences represent real-life challenges when it comes to the BCG signal applications since they present different properties than the perfect conditions assumed in the previous works. This fact makes this work a novelty and boosts its adaptability and use case for physical applications.

This work began with a temporal and spectral analysis of the resulting BCG signals. This was followed by unsupervised learning to understand the patterns available in the presented dataset. Furthermore, the different parameters of the segmentation were explored and rigorously determined. Next, two-label classification was carried out to create flags indicating the opportunity of measuring cardiac and respiratory activities. Finally, two different methods of multi-label classification were proposed. The sample-by-sample classification showed more promising results compared with frame-by-frame classification.

## Figures and Tables

**Table 1:** True Positive Rate (TPR) and Positive Predicted Value (PPV) confusion matrix for cardiac activity detection.

True Class		PPV		TPR
	CAD	NoCAD	CAD	NoCAD
CAD	95%	15%	98%	2%
NoCAD	5%	85%	33%	67%

**Table 2:** TPR and PPV confusion matrix for respiratory detection.

Predicted Class	PPV			TPR
	RAD	NoRAD	RAD	NoRAD
RAD	90%	17%	95%	5%
NoRAD	10%	83%	28%	72%

**Table 3:** PPV confusion matrix by frame-by-frame classification.

Class	Normal	Coughing	Post-Coughing	Holding Breath	Expiration	Movement	Others
Normal	94%		28%	30%	33%	21%	45%
Coughing	6%	100%	4%	10%	11%	23%	10%
Post-coughing			62%				
Holding breath			3%	57%			
Expiration			<1%	1%	56%		
Movement			3%	3%		55%	
Others			<1%			2%	45%

**Table 4:** TPR confusion matrix by frame-by-frame classification.

Class	Normal	Coughing	Post-Coughing	Holding Breath	Expiration	Movement	Others
Normal	71%		22%	2%	1%	3%	1%
Coughing	24%	38%	16%	4%	1%	17%	1%
Post-coughing			100%				
Holding breath			35%	65%			
Expiration			16%	4%	80%		
Movement			21%	2%		77%	
Others			32%			21%	47%

**Table 5:** TPR confusion matrix for sample-by-sample classification.

Class	Normal	Coughing	Post-Coughing	Holding Breath	Expiration	Movement	Others
Normal	98%		1%	<1%	<1%		
Coughing	1%	92%	2%	1%		4%	
Post-coughing	1%	2%	96%		<1%	1%	
Holding breath	31%	<1%		68%	<1%		
Expiration				7%	93%		
Movement	4%		1%			86%	9%
Others			7%				93%

**Table 6:** PPV confusion matrix for sample-by-sample classification.

Class	Normal	Coughing	Post-Coughing	Holding Breath	Expiration	Movement	Others
Normal	96%		3%	7%	13%		
Coughing	<1%	96%	1%	3%		7%	
Post-coughing	<1%	4%	95%		3%	4%	
Holding breath	3%	<1%		87%	<1%		
Expiration				3%	84%		
Movement	1%		<1%			90%	23%
Others			1%				77%

## Abbreviations

The following abbreviations are used in this manuscript:

AIC	Akaike Information Criterion
BCG	Ballistocardiogram
BIC	Bayesian Information Criterion
DFT	Discrete Fourier Transform
EMD	Empirical Mode Decomposition
FOS	Fiber Optic Sensor
GMM	Gaussian Mixture Model
IoMT	Internet of Medical Things
KNN	K-Nearest Neighbors
SFM	Spectral Flatness Measure
SC	Spectral Centroid
TPR	True Positive Rate
PPV	Positive Predicted Value
WT	Wavelet Transform
